# Disrupting links between poverty, chronic stress, and educational inequality

**DOI:** 10.1038/s41539-023-00199-2

**Published:** 2023-11-20

**Authors:** Madeline B. Harms, Sherona D. Garrett-Ruffin

**Affiliations:** 1 Department of Psychology, University of Minnesota Duluth, Duluth, USA; 2 Department of Psychology, Bowling Green State University, Bowling Green, USA

**Keywords:** Education, Human behaviour

## Abstract

The income-achievement gap is a significant and stubborn problem in the United States, which has been exacerbated by the Covid-19 pandemic. In this article, we link two emerging literatures that have historically been disparate: the neurobiology of poverty as a form of early life stress, and research on educational policies with the potential to reduce SES-based disparities in academic achievement. In doing so, we (1) integrate the literature on poverty-related mechanisms that contribute to early life stress, alter neurobiology, and lead to educational inequities, and (2) based on this research, highlight policies and practices at the school/classroom level and broader structural level that have the potential to address the problem of inequity in our educational systems. We emphasize that educational inequity is a systemic issue, and its resolution will require coordination of local, state, and national policies.

## Introduction

Over 12 million children in the United States live in poverty^1^, which can be defined as having resources below the average family that results in an inability to fully participate in society^2^. Children living in poverty may experience a plethora of disruptive life events and circumstances--for example, lower-quality prenatal care, economic strain, frequent moves, higher rates of illness, food insecurity, neighborhood violence, malnutrition and greater exposure to pollution and toxins^3–6^. Children in poverty are also more likely to be exposed to high levels of parental stress, increasing risk for negative parenting practices^7,8^. Lack of parental resources to buy books and other educational materials may result in a lack of cognitive stimulation in the home^9,10^, and lack of time for parents to engage in conversation with children may result in lower language exposure^11^. In addition to exposing families to numerous stressors, poverty can undermine family support and other processes that would otherwise enable positive coping with such stressors^8^.

All these factors contribute to socioeconomic disparities in academic achievement. These disparities in turn contribute to lower occupational attainment and intergenerational cycles of poverty. In the past two decades, scientific understanding of the mechanisms contributing to the income-achievement gap has increased dramatically. A growing body of evidence suggests that growing up in poverty contributes to *cumulative risk exposure*—resulting in chronic stress which impacts neurocognitive development in ways that tend to hinder academic performance^12–14^. Although a low family income, in and of itself, is not necessarily stressful to children, poverty-level income often results in multiple contextual stressors and decreases families’ abilities to cope with those stressors. We use the term “poverty-related stress” to refer to this overall context of poverty. Building on previous reviews discussing links between socioeconomic status, neural circuitry, and academic readiness^15^, our aims in this article are: (1) to integrate the literature on poverty-related mechanisms that contribute to early life stress, alter neurobiology, and contribute to educational inequities, and (2) to highlight policies and practices at the classroom, school and broader systemic levels designed to address educational inequity that map onto scientific knowledge regarding the effects of chronic stress on brain development. Although child poverty is a worldwide problem, our policy recommendations will focus on the United States due to its unique economy and societal structure. We expect most aspects of this review to generalize to other high-income countries, but we acknowledge that children in low- and middle-income countries face additional challenges not covered here.

## Poverty and early life stress

There is strong empirical support for the notion that poverty gets “under the skin,” meaning that the experience of poverty can lead to long-lasting biological changes in individuals^16^. Experiencing stress that is chronic and severe at early ages appears to result in neuroendocrine profiles that bias the developing nervous system toward responding to events in reactive and defensive ways^17^, in contrast to a reflective and regulated state that would facilitate academic learning. For example, childhood poverty is linked to both increased activity in threat-detecting brain circuitry and decreased activity in self-regulatory circuitry^18^. When a child is extra vigilant to potential threats in the environment at school (e.g., remarks from a teacher or classmate, or a sense of being “behind” her peers) and lacks the tools to reason through and regulate that sense of threat, she is unlikely to be able to focus optimally on learning in the classroom. Importantly, the same neural mechanisms may confer children growing up in adverse circumstances with “hidden talents” that facilitate their ability to function in harsh, unpredictable environments, including enhanced social perception, attention shifting, and creativity^19^, a point we will return to under policy recommendations.

The influence of chronic stress during childhood and adolescence (relative to later periods of life) may be exacerbated due to rapid changes in brain circuitry and maturation of the neuroendocrine system during development^20^. Chronic stress influences brain development via its effects on the Hypothalamic-Pituitary-Adrenal (HPA) axis, which regulates the body’s response to environmental demands via the release of the hormone cortisol from the adrenal gland. During periods of high stress, cortisol production is increased to help the organism deal with the stressor. However, if stress is prolonged and severe, both daily cortisol production and cortisol responses to an acute stressor may be disrupted^21–23^, and these disruptions are associated with health, cognitive, and behavioral problems^24^. Both early childhood and adolescence appear to be periods during which the HPA system shows heightened susceptibility to environmental influence^22^. This means that chronic and severe stress experienced during these developmental periods may be especially detrimental, tuning the nervous system to deal with stress in dysregulated ways. On the other hand, buffers or protections from stress during these times may be especially beneficial—in particular, adolescence may be a window of opportunity for consequences of previous exposure to stress to be mitigated, if positive environmental supports are present^25^.

At the neurocircuitry level, chronic stress resulting from poverty appears to impair the function of two major brain systems: (1) circuitry that allows individuals to regulate thoughts and emotions—broadly called executive function and subserved by prefrontal cortex—and (2) circuitry that facilitates detection of important sources of threat and reward in the environment (consisting of amygdala, ventral parts of prefrontal cortex, and their connections). Imbalance in detection of threat and reward may in turn impair the development of basic associative learning mechanisms and their neural correlates, including orbitofrontal cortex and anterior cingulate cortex^26,27^, as well as basal ganglia circuitry^28^. Furthermore, threat-detection circuitry, particularly the amygdala, activates the HPA system, facilitating the physiological stress response^29^. These inter-related brain systems of executive function and threat/reward processing likely mediate the link between socioeconomic status (SES) and academic achievement, given that academic learning depends upon a sense of safety in the classroom (low threat vigilance), and both the motivation (anticipation of reward) and ability to work towards long- term goals (executive function). For example, executive function has been shown to mediate the link between family SES in elementary school and middle school academic performance^30^.

Importantly, early life stress seems to simultaneously increase neural sensitivity to threat and decrease the ability to detect potential reward. For example, childhood poverty predicts higher amygdala and medial prefrontal cortical responses to threatening faces^31^, which may be mediated by systemic inflammation^32^. These patterns of neural response suggest heightened vigilance to threatening stimuli among some individuals who experienced poverty as children (and may also be associated with inflammation, a threat to physical health). Reward systems are also impacted by early life stress: children who experienced multiple forms of chronic stress show lower neural activation to reward in orbitofrontal brain regions during an associative learning task, along with reduced exploratory learning^26^, and a review of early childhood stress suggests lower ventral striatum function across several human and animal studies^28^. Finally, a recent comprehensive review of neuroimaging work suggests that childhood poverty/low SES alters the structure and function of brain circuitry involved in a broad number of systems necessary for adaptive functioning, including executive attention, decision-making, emotion regulation, and salience evaluation and interpretation^18^. Notably, although we have outlined reasons to predict links between neural threat and reward processing and academic learning, there is little existing research that explicitly investigates these associations.

Altered development of self-regulation and salience (i.e., threat and reward) detection systems have important implications for a child’s ability to function in a traditional Western school environment. A recent detailed review of implications of stress physiology for educational inequity outlines many of these implications: that the school year can be considered a period of adaptation (e.g., new teacher, classroom, classmates) that places high demands on a child’s stress response systems; that children from disadvantaged SES and demographic groups are likely to experience more stressors and be more affected by them due to their life history; and that teacher behavior and classroom quality can exacerbate or lessen these demands^33^. In the next section, we outline reasons why economically disadvantaged children may experience lower-quality educational environments.

## Poverty and unequal access to quality education

Given that children living in poverty already experience numerous stressors, it is important to consider how pedagogical practices and the school environment may create additional chronic stress that undermines learning, further contributing to disparity in academic outcomes. Low-income students experience higher suspensions and expulsion rates, have less access to extracurricular activities, and may experience higher rates of bullying^34,35^. In addition, schools with a high proportion of students in poverty may have higher teacher turnover rates and fewer highly qualified teachers (i.e., less teaching experience and lower teaching effectiveness)^36–38^. Teachers in such schools also tend to experience higher levels of stress and burnout, which in turn negatively impacts student-teacher relationships, further reducing teacher effectiveness^39^. Although it is difficult to disentangle the school environment as a source of chronic stress from other factors in children’s lives, a recent study found that being bullied at school was linked to higher hair cortisol, which was in turn associated with poorer executive function^40^. This indicates that the school environment can serve as a source of chronic stress for children and have effects on academic skills.

Children living in poverty are also more likely to be impacted by inequitable school policies and negative social events. These children are often stereotyped as unmotivated and lacking aspiration^37^ and are targets of discrimination manifested from negative practices, attitudes and institutional policies^41^. For example, during the COVID-19 pandemic, children living in poverty were disproportionately likely to experience less access to the internet, less access to school lunch programs, higher levels of food insecurity, and more unsupervised time^42–44^. Furthermore, although surveys indicate that regardless of income level, students perceive schools to be unsafe due to fear of school shootings and other forms of violence^45,46^, students in low-income schools are more likely to experience violence prevention measures such as random searches, metal detectors and police presence^47^. Thus, worries about school violence coupled with the violence prevention measures described above could lead to “anticipatory trauma” resulting in chronic stress^48^. Conversely, when schools can provide safety and support, children living in poverty are likely to benefit disproportionately^49^ and are able to access the economic opportunities that come with having a high-quality education.

## Neuroscience-based educational practices

As discussed earlier, research indicates that poverty is a source of chronic stress that contributes to executive function deficits, emotional dysregulation, and disruptions in salience detection^18^, which in turn impact both basic and academic learning processes^33^. To be effective in school environments, students therefore must be able to identify and manage (i.e., regulate) emotions, and understand how emotions impact decision making. Given the connection between stress, learning, and emotion, and the disproportionate effects of early life stress on executive processes, it may be particularly important to address the emotive elements of learning for children in poverty by (1) reducing potential sources of stress inadvertently embedded in the curriculum and pedagogical practices and (2) bolstering children’s emotion regulatory skills. Fortunately, there are existing trends in education, such as social emotional learning practices, that aim to address both these issues.

Scientific understanding of the connection between executive functions (EF) and children’s ability to learn in school has increased dramatically in the past decade. For example, in a study of pre-K children, both EF (working memory, inhibitory control) and emotion regulation (tested with a delay of gratification task) have been shown to predict school readiness, with EF predictive of academic readiness and emotion regulation linked to social-emotional readiness^50^. Furthermore, in this study early social-emotional readiness predicted later academic readiness, suggesting that social-emotional skills must be in place for academic learning to be optimized. More broadly, there is growing scientific consensus that aspects of EF, emotion regulation, and skills needed for school readiness are multidimensional and likely to be reciprocally linked^51^.

Related to this emerging research, in recent years, a set of curricular practices called Social Emotional Learning (SEL) has gained traction in the U.S. and worldwide^52^. Endorsing this emphasis, a recent UNESCO global report on the future of education noted that “Education policy and practice focusing on academic performance rather than balancing it with social and emotional competencies, has led to a decline in human and societal flourishing”^53^ The goal of SEL is to help children develop the “knowledge, attitudes, and skills necessary to understand and manage emotions, set and achieve positive goals, feel and show empathy for others, establish and maintain positive relationships, and make responsible decisions”^54^. Some assumptions behind this method are that meaningful academic learning in a school setting is only possible when students are motivated, self-regulated, and able to connect with their peers; and that the purpose of schooling goes beyond pure academic learning--formal education should also develop students’ interpersonal skills that they will need for work and life.

Approaches to SEL must be tailored to children’s developmental level. Young children benefit more from SEL programs that focus on skill development, skill supplementation and skill revision, as compared to elementary and high school students who benefit more from a combination of skill-based and mindset-based SEL programming^55^. Given young children’s developing understanding of perspective taking and mixed emotional states, preschool SEL programs provide students with opportunities to practice sharing, cooperation and learning via modeling, sociodramatic play, and teacher feedback^56^. In elementary school, specific practices build on developing social and academic skills, ranging from Morning Meetings, a structured space for students to check in with their emotions and with each other at the start of the school day; to forms of inquiry-based learning, such as “Discover, Discuss, Demonstrate.” Adolescents are confronted with more complex psychosocial developmental tasks, such as developing identity, autonomy, acceptance from peers, competence, and goal attainment^57^. Effective adolescent SEL programs have been found to build on students’ desires for status and respect by providing opportunities for them to use SEL skills and concepts in meaningful ways, such as serving the community and leading school discussions around topics of developmental interest, such as teen-age pregnancy, youth violence and smoking^58–60^.

A number of studies find that SEL programs, when implemented well, have positive impacts both on growth in the skills targeted and on academic outcomes^52,60,61^. Multi-country meta-analytic studies also indicate largely positive effects of SEL programs on both academic outcomes^62^ and long-lasting impacts on socio-emotional skills and high school graduation rates^63^. There is also intriguing evidence that SEL may have positive impacts on children’s stress physiology. In a randomized trial, considered the gold-standard for program efficacy research, Schnonert-Reichl and colleagues^52^ found that 4th-5th grade children who received an SEL program that included mindfulness training (MindUP) showed steeper diurnal cortisol slopes at post-test (indicative of healthier HPA axis function), as well as improved outcomes in social-emotional functioning, self-reported school self-concept, and teacher-reported math achievement, relative to a control group of children who received a social responsibility training program. In another recent study, behavior improvements in a group of low-income kindergartners enrolled in an SEL program (PATHS) were observed relative to an active control condition, which were sustained over time, even a year after the program ended^64^. Interestingly, children with lower heart rate variability (HRV) at the start of intervention--which usually reflects poor emotion regulation--were more likely to show sustained behavioral benefits from the program a year after it ended, suggesting less sensitivity to the withdrawal of a supportive environmental asset.

These results indicate that stress physiology can interact with behavioral interventions in complex ways, emphasizing the need for more research integrating both biological and behavioral outcome measures^33^. Although there is robust evidence that SEL contributes positively to the social and academic development of both middle class and disadvantaged children^61^, more research is also needed to determine the mechanisms of influence--in particular, whether SEL contributes to positive changes in socio-emotional skills and learning via biological changes in stress physiology and/or neural function. For example, do SEL programs improve biological correlates of emotion regulation (vagal tone, prefrontal connectivity to the limbic system) and salience detection (neural activation to anticipated reward/less hypervigilance to threat)? In other words, we have yet to determine the extent to which SEL can “get under the skin” and promote resistance to *physiological* consequences of stress resulting from poverty. This knowledge is important because to implement the most effective interventions, scientists and practitioners should understand the mechanisms through which they operate. For example, an intervention that positively alters physiology *and* behavior would be expected to have a more long-lasting and generalizable impact than one that influences behavior, but not underlying neurophysiology.

Further research is also needed to identify factors that influence the efficacy of SEL programs. Such programs often involve multiple components, such as activities to foster positive classroom community, mindfulness exercises, and changes in methods of instruction (e.g., greater emphasis on active or self-directed learning). We cannot currently determine whether one of these components makes most of the difference, or whether the holistic integration of all facets is needed (i.e., the whole is greater than the sum of its parts). In this regard, it may be telling to examine instances where SEL-related curriculum changes were not successful. For example, one multi-site study found no improvement in mental health outcomes after an 8-week mindfulness program among middle school students; in fact, the program appeared to increase anxiety among some subgroups with low baseline mental health concerns^65^. Explanations suggested by the authors included lack of research on the developmental trajectory of mindfulness in early adolescence, low at-home compliance with the program, and variation in instructor experience. In contrast, in a small study of predominantly Hispanic/Latino at-risk students at an alternative high school^66^, students who completed a similar mindfulness program showed reductions in anxiety, perceived stress, and depression relative to a control group that completed a substance abuse prevention program. The authors suggest factors that were crucial to success, including establishing a physical environment where students felt safe (gym versus classroom), establishing trust with the instructor during unstructured times, and inviting versus requiring student participation, giving students a sense of agency^66^. Results from larger meta-analyses and randomized control trials of mindfulness interventions in adolescents have been mixed, with little evidence for unambiguously positive outcomes^67,68^. Mixed success of isolated mindfulness programs, in contrast to mostly positive effects of broader SEL programs, suggest that integrated curricula with classroom teachers (who may serve as safety figures to children) leading activities may be key to these positive outcomes. This would be consistent with the notion that supportive adults can buffer physiological stress responses in children^69^, but this remains to be tested in the school environment.

Given increasing racial and ethnic diversity in the U.S. population, cultural sensitivity is also important to consider. Jagers, Rivas-Drake, & Williams^70^ discuss the notion of “transformative SEL,” which would foster “critical citizenship” in addition to the broad goals of SEL programming. They contrast transformative SEL with more traditional personally responsible or participatory forms, which could actually undermine aspects of identity development for ethnic/racial minority or immigrant children by facilitating assimilation and/or acculturation, implicitly endorsing the superiority of majority group norms. Transformative SEL, in contrast, would equip students with knowledge and skills to challenge unjust norms they encounter. Transformative SEL practices aim to promote positive cultural identity and a sense of agency/purpose, which have been linked to more positive stress profiles^70–72^ that may support resilience.

Finally, better attempts to leverage potential cognitive advantages that low-SES children may possess, including social perception, attention shifting, and creativity, have the potential to mitigate SES-based educational disparities. These might take the form of increased team or group work, an emphasis on applying learned material to real-world problems, and incorporating oral or narrative learning and assessment strategies^19^. These suggested strategies need to be tested through empirical research.

## System-level strategies to reduce the effects of poverty on academic achievement

To reduce SES-based disparities in academic achievement, it is our view that multifaceted and structural approaches that change policies at the national, state and district levels, as well as addressing the school environment, home environment and community environment, are needed. Poverty reduction strategies must address the structural causes of poverty. Sadly, due to political stalemate, little progress on this issue has been made in the past 30 years, with the exception of some temporary measures taken during the Covid-19 pandemic (e.g., expanding the Child Tax Credit). At the national, state and district level, a restructuring of funding policies is necessary to ensure that schools receive necessary resources to support low-income students. For example, the practice of funding schools using property taxes is insufficient in higher poverty areas and is arguably unconstitutional because such funding practices lead to inherently unequal schools in terms of resources and opportunities^73^. To reduce the achievement gap among racially minoritized children, policies are needed to reduce not only poverty, but also structural racism. For example, Black, Latinx, and Native American students experience more suspensions and exclusions as compared to other demographic groups, although they do not display more problematic behavior^74^. In addition, paid school lunch policies in many states create unnecessary burdens to food access that hinder learning. Finally, children living in poverty need greater access to preventive, curative and diagnostic healthcare. It is crucial to acknowledge the need for these broad changes to school funding structures, disciplinary practices, access to physical and educational resources, and healthcare systems as a foundation that is needed to facilitate the school and classroom-level changes we recommend.

## Discussion

The income-education gap is a difficult problem, and schools and communities have unique structural challenges in addressing this issue. While still in the early stages, researchers are beginning to use the neuroscience of stress to inform teaching and learning practices that may contribute to equalizing educational opportunity. Further research is needed to determine which aspects of current intervention programs are most effective (e.g., explicit training of emotion regulation skills, cultivation of caring teacher-child relationships, classroom community building, critical citizenship), how these interventions programs may impact children’s physiological responses to stress and contribute to their overall health, and how neural responses affected by chronic stress (e.g., threat and reward detection) impact academic learning. Our understanding of these mechanisms will be aided by identifying the indirect pathways through which poverty-related stress affects academic achievement (e.g., via family conflict, cognitive deprivation, physical inflammation, etc.). As various mechanisms are identified, more targeted interventions can be developed to address these specific issues. In addition, further research is needed on potential impacts of genetics and gene-environment correlation on both susceptibility to stress and academic achievement^75,76^. In part due to the fallout from the Covid-19 pandemic, the current state of education policy in the U.S. is at a turning point, presenting an exciting opportunity for researchers and practitioners to integrate stress neurobiology into the curriculum and positively influence children’s overall development.

### Reporting summary

Further information on research design is available in the Nature Research Reporting Summary linked to this article.

### Supplementary information

Reporting Summary

## Data Availability

No primary data was used in writing this manuscript.
